# Collagenous Gastritis Masquerading as Eosinophilic Gastritis

**DOI:** 10.14309/crj.0000000000000527

**Published:** 2021-02-22

**Authors:** Jeremy Klein, Benjamin J. Wilkins, Ritu Verma, Amanda B. Muir

**Affiliations:** 1 Lewis Katz School of Medicine at Temple University Hospital, Philadelphia, PA; 2 Division of Gastroenterology, Hepatology and Nutrition, The Children's Hospital of Philadelphia, Philadelphia, PA; 3 Department of Pathology and Laboratory Medicine, The Children's Hospital of Philadelphia, Philadelphia, PA; 4 Department of Pediatrics, University of Chicago, Chicago, IL; 5 Department of Pediatrics, Perelman School of Medicine, University of Pennsylvania, Philadelphia, PA

## Abstract

A 11-year-old boy presented to the gastroenterology clinic after a 5-month history of fatigue, pallor, intermittent abdominal pain, and iron-deficiency anemia. Although the initial upper endoscopy was visually normally, the histological assessment was suggestive of eosinophilic gastritis. After multiple scopes and failed therapies, histologic analysis revealed a focus of thickened subepithelial collagen deposition suggestive of collagenous gastritis. A retrospective review of gastric biopsies using Gomori trichrome stain revealed previously unappreciated collagen deposition. This case report illustrates the benefit of performing trichrome stain on gastric biopsies in the setting of persistent or isolated gastric eosinophilia or iron deficiency anemia.

## INTRODUCTION

Collagenous gastritis (CG) is a rare gastrointestinal (GI) disorder characterized by anemia or abdominal pain, stomach-specific inflammation, and subepithelial collagen deposition.^1^ There have been approximately 25 reported pediatric cases since the first description of CG by Coletti and Trainer in 1989.^2^ At present, reports distinguish between a pediatric phenotype consisting of only gastric pathology with upper GI symptoms, and an adult phenotype with simultaneous collagenous colitis often associated with immune dysregulation. In both phenotypes, CG is characterized by the presence of patchy subepithelial collagen bands (>10 μm) along with lymphocytic or eosinophilic inflammatory infiltrates in the gastric mucosa.^3^ CG pathogenesis and etiology remain poorly understood because of the rarity of cases and the heterogeneity of inflammation and collagen deposition. Presentation often involves severe anemia, believed to be caused by entrapment of gastric capillaries by collagen leading to hemorrhage.^4^ Current treatment regimens are not therapeutic but rather are meant to treat ongoing blood loss; in the pediatric phenotype, patients are dependent on long-term iron supplementation.^5^ In this report, we describe an 11-year-old patient who initially presented with symptoms of severe anemia. This patient had a tortuous course from presentation to diagnosis but was ultimately diagnosed with CG. After histological re-evaluation of all gastric biopsies, we determined the patient had CG from the onset of care that histologically mimicked eosinophilic gastritis (EG).

## CASE REPORT

A 11-year-old boy presented with 3 months of increasing fatigue, intermittent abdominal pain with cramping, occasional nausea, and pallor. Hematologic workup revealed microcytic iron deficiency anemia (hemoglobin 4.1 g/dL, mean corpuscular volume 62 fL, absolute reticulocyte count 74.8 thou/μL, and ferritin of 1.9 ng/mL). The differential was broad including hematologic malignancy, inflammatory bowel disease, celiac disease, helicobacter pylori infection, and excessive milk intake. Initial workup included the following tests, which were all within normal limits: calprotectin, thyroid-stimulating hormone, urinalysis, fecal occult blood, C-reactive protein, thoracic x-ray, and complete metabolic panel. Initial celiac testing showed borderline antiendomysial antibodies and normal tissue transglutaminase, but repeat testing was normal. He was treated with 10 cc/kg of packed red blood cell transfusion followed by 3 months of ferrous sulfate 325 mg twice daily. At discharge, he was given a presumptive diagnosis of iron deficiency anemia because of excess milk intake, and he was instructed to decrease consumption. His anemia returned after cessation of oral iron, and he underwent esophagogastroduodenoscopy (EGD) which was visually normal, and histologically, there was no evidence of intestinal pathology, celiac disease, or Helicobacter pylori save for mild chronic active gastritis.

Two months later, his hematologic studies indicated recurrence of anemia, and an upper and lower endoscopy was performed. Visually, there was marked nodularity of the stomach noted. Histologically, there was a patchy increase in lamina propria eosinophils (up to 40 per high power field) with focal eosinophilic microabscesses and mild, chronic inflammation with lymphoid follicle. Terminal ileum and colon were histologically normal. Stool testing confirmed the absence of ova or other parasitic infection.

**Figure 1.**
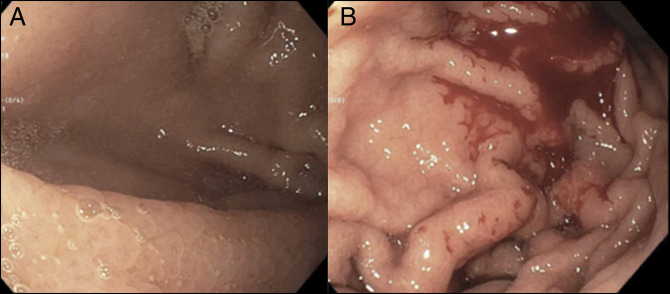
Endoscopic appearance: Representative photographs from endoscopy at 12 years 0 months both (A) before and (B) after biopsy. There is nodularity and edema of the gastric mucosa with distinct granularity.

He was offered treatment with prednisone, but because this may be a food allergy–driven process, the patient decided to stop wheat consumption for 3 months, and an EGD was repeated.^6^ The endoscopy again showed nodularity with diffuse friability and granularity in the gastric fundus, body, and antrum (Figure 1). Histologic analysis showed chronically inflamed antral and oxyntic type gastric mucosa with moderate eosinophilia (65/hpf), and patchy subepithelial collagen deposition (up to 100 μm in thickness) confirmed by Gomori trichrome stain. Follow-up gastric biopsies after a trial of topical budesonide revealed continued subepithelial collagen deposition (65 μm) with mucosal eosinophilia (65/hpf).^7^ Currently, the patient remains under care for the management of his anemia, growth development, and potential long-term effects of CG. He is managed on oral iron and is not eliminating any foods from his diet.

## DISCUSSION

We describe a case report of CG initially diagnosed with EG. Both EG and CG present with nonspecific GI symptoms (including vomiting, pain, and diarrhea), anemia, and nodular stomach on endoscopy and both conditions can have marked eosinophilia of the stomach. However, therapy varies underscoring the need for accurate diagnosis.

This case demonstrates the histologic evolution of disease; first, there was inactive gastritis, then eosinophilia, and finally marked collagen deposition. Upon re-examination, we performed Gomori trichrome on all gastric biopsies. This revealed early development of CG and focal/minor collagen deposition-even in the first biopsy (maximum subepithelial collagen thickness of 34 μm with 5% of total tissue surface found with subepithelial collagen bands > 10 μm). Trichome staining also confirmed collagen deposition in the second biopsy which was originally found most consistent with EG (50/hpf). Biopsies from the patient's ensuing EGDs all showed collagen deposition (ranging from 60 to 100 μm involving 70%–90% of the tissue surface).

**Figure 2.**
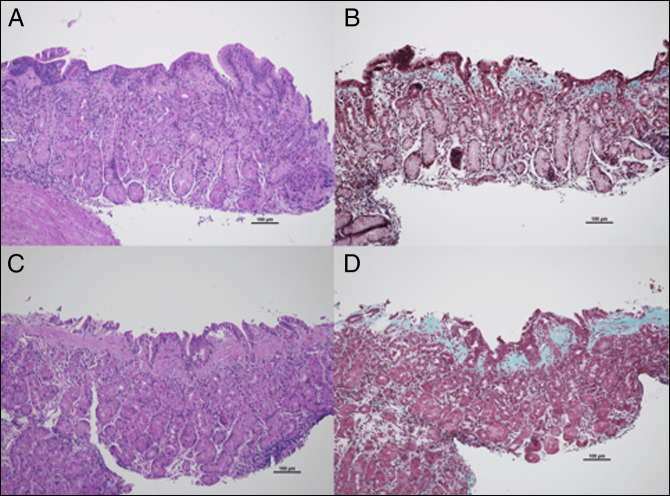
Representative gastric mucosa from patient at age. Initial diagnosis of chronic inactive gastritis at 11 year 4 months (not shown). (A and B) gastritis with increased eosinophils at 11 year 8.5 months, (C and D) collagenous gastritis at 12 year 0 months onward. Retrospective review showed at least focal features of collagenous gastritis in all specimens (hematoxylin and eosin stain for A, C, and Gomori trichrome for B, D).

Consistent with other published case series on CG, collagen deposition is often not increased enough or overlooked on hematoxylin and eosin stain alone to prompt additionally staining, and there is often increased eosinophilia noted in the lamina propria in CG.^3^ The patient's first biopsies with hematoxylin and eosin staining revealed very minimal subepithelial deposition, not impressive enough to suggest trichrome staining. However, through this case, we are able to witness, histologically and endoscopically, how the collagen deposition increases over time (and how the patient evolved from a visually normal-appearing stomach to marked nodularity, perhaps lending a clue to the natural history (Figure 2). It is also important to acknowledge the limitations of biopsy sampling variation and the inherent patchy nature of early collagen deposition and to consider increased number of biopsies and alternative staining when disease presentation and histology do not correlate.^8^

This case demonstrates the importance of performing Gomori trichrome stain on pediatric patients with suspected GI pathology in persistent EG or recurrent iron deficiency anemia. Although both conditions are rare, especially in pediatrics, keeping a broad differential in the setting of eosinophilic inflammation (drug reaction, allergy, infection, and CG) will prevent unnecessary dietary changes or steroid therapy used in eosinophilic conditions. Finally, despite the absence of more definitive GI symptoms, gastroenterologists should consider early endoscopic evaluation, biopsy, and trichrome staining for patients with EG or persistent unexplained iron deficiency anemia.^3^

## DISCLOSURES

Author contributions: J. Klein, R. Verma and AB Muir wrote the manuscript. B. Wilkins performed the pathological analyses. AB Muir is the article guarantor.

Financial disclosure: None to report.

Informed consent was obtained for this case report.
